# Short-Course or Total Neoadjuvant Chemotherapy in Resectable and Borderline Resectable Pancreatic Cancer - Current Status and Future Perspectives

**DOI:** 10.3389/fsurg.2022.839339

**Published:** 2022-04-25

**Authors:** Knut Jørgen Labori

**Affiliations:** ^1^Department of Hepato-Pancreato-Biliary Surgery, Oslo University Hospital, Rikshospitalet, Oslo, Norway; ^2^Institute of Clinical Medicine, University of Oslo, Oslo, Norway

**Keywords:** pancreatic cancer, neoadjuvant chemotherapy, precision medicine, surgery, Response Evaluation Criteria

## Abstract

Neoadjuvant therapy improves overall survival compared with a surgery-first approach in patients with borderline resectable pancreatic cancer (BRPC). Evidence of higher quality is required to determine whether neoadjuvant therapy has potential benefits and improves survival for patients with resectable pancreatic cancer (RPC). Most randomized controlled trials (RCTs) have explored short-course neoadjuvant chemotherapy (SNT), but total neoadjuvant chemotherapy (TNT) is now the experimental arm of ongoing RCTs. This article reviews the current status of SNT and TNT in RPC and BRPC, and provides perspectives of future challenges and research directions in this field.

## Introduction

Pancreatic ductal adenocarcinoma is a lethal malignancy, and surgical resection remains the only potential for cure. Data from population-based registries show resection rates of 13–21.7% (1). Resectable pancreatic cancer (RPC) is defined according to the National Comprehensive Cancer Network classification (NCCN) as the absence of distant organ or lymph node metastases; no tumor contact with the superior mesenteric/portal vein, or ≤ 180° contact with either vein without vein contour irregularity, and no tumor contact with the coeliac axis, common hepatic, and superior mesenteric artery (2). Borderline resectable pancreatic cancer (BRPC) is determined by limited involvement of the superior mesenteric/portal vein or arterial axis (2). Currently, a surgery-first approach is the universally accepted standard practice for RPC, followed by 6 months of adjuvant chemotherapy, preferably with mFOLFIRINOX (modified 5-fluorouracil with leucovorin, irinotecan, and oxaliplatin) in fit patients (3, 4). For BRPC contemporary approaches have focused on neoadjuvant chemotherapy with the goal of improving overall survival (2, 5).

Neoadjuvant chemotherapy is sometimes used for RPC, especially in patients with high-risk features, i.e., radiographic findings are suspicious but not diagnostic for metastatic disease, potentially reversible performance status or comorbidity profile that is not currently appropriate for major surgery, or a carbohydrate antigen 19-9 (CA19-9) level suggestive of metastatic disease (2). For patients with RPC, who are fit to undergo upfront surgery, neoadjuvant therapy is considered best to be managed within the boundaries of a clinical trial (2). A major criticism of published adjuvant RCTs in RPC is that these trials only included patients who recovered from a successful operation, and in some trials restaging with a CT and CA19-9 was performed to exclude patients with early recurrence (4, 6–11). Most retrospective studies on neoadjuvant therapy in RPC only include patients undergoing a successful operation and also suffer from selection bias (10). Currently, several RCTs are evaluating the effect of neoadjuvant chemotherapy in RPC and BRPC (Table 1). The data from these trials will expand the knowledge on treatment sequencing and survival in RPC and BRPC, and better delineate the completion rates of all parts of multimodal treatment.

**Table 1:** Randomized controlled trials of neoadjuvant therapy in resectable and borderline resectable pancreatic cancer.

**Trial/Months**	**1**	**2**	**3**	**4**	**5**	**6**	**7**	**8**	**Status**
ALLIANCE 021806 (12)	FOLFIRINOX				Surgery		FOLFIRINOX		Ongoing
(R, *N* = 352)	Surgery						FOLFIRINOX		
PREOPANC-3 (13)	FOLFIRINOX				Surgery		FOLFIRINOX		Ongoing
(R, *N* = 378)	Surgery						FOLFIRINOX		
NORPACT-1 (14)	FOLFIRINOX		Surgery		FOLFIRINOX				Accrual
(R, *N* = 140)	Surgery				FOLFIRINOX				completed
PANACHE 01 (15)	FOLFIRINOX		Surgery		Adjuvant chemotherapy at the discretion of medical team				Ongoing
(R, *N* = 168)	FOLFOX		Surgery		Adjuvant chemotherapy at the discretion of medical team				
	Surgery				Adjuvant chemotherapy at the discretion of medical team				
SWOG S1505 (16)	FOLFIRINOX			Surgery		FOLFIRINOX			Published
(R, *N* = 102)	Gem/Nab-pac			Surgery		Gem/Nab-pac			
ESPAC-5F (17)	FOLFIRINOX		Surgery		5 FU/FA or Gem at the discretion of medical team				Published
(R, *N* = 88)	Gem–Cap		Surgery		5 FU/FA or Gem at the discretion of medical team				(abstract)
	Cap-Radiotherapy		Surgery		5 FU/FA or Gem at the discretion of medical team				
	Surgery				5 FU/FA or Gem at the discretion of medical team				
PREOPANC-2 (18)	FOLFIRINOX				Surgery				Accrual
(R/BR, *N* = 368)	Gem/Gem–Radiotherapy			Surgery		Gem			completed
PREOPANC-1 (19)	Gem/Gem–Radiotherapy			Surgery		Gem			Published
(R/BR, *N* = 246)	Surgery					Gem			
JSAP (20, 21)	Gem-S1		Surgery		S-1				Published
(R/BR, *N* = 362)	Surgery				S-1				(abstract)
NEONAX (22)	Gem/Nab-pac		Surgery		Gem/Nab-pac				Ongoing
(R, *N* = 166)	Surgery				Gem/Nab-pac				
PACT-15 (23)	PEXG			Surgery		PEXG			Published
(R, *N* = 88)	Surgery		PEXG/capecitabine						
	Surgery		Gem						
Wisconsin perspective (24)	Neoadjuvant chemotherapy at the discretion of medical team				Chemoradiotherapy	Recovery	Surgery		Published
(R/BR, *N* = 89)	(16 weeks)				(5.5 weeks)	(4 weeks)			

*Non-surgical preoperative therapy includes neoadjuvant chemo(radio)therapy for 2, 3, or 4 months in the randomized controlled trials. Surgery is performed 3–6 weeks after completion of chemotherapy or a minimum of 4 weeks after chemoradiotherapy*.

## Outcomes of Neoadjuvant Treatment in Comparative Studies

A recent meta-analysis by van Dam et al., of randomized clinical trials (RCTs), found that neoadjuvant therapy improves survival compared with upfront surgery in patients with BRPC (10). However, more evidence is required on whether neoadjuvant therapy improves survival for patients with RPC (10). Long-term results of the PREOPANC-1 trial, the largest RCT to date comparing neoadjuvant chemoradiotherapy with upfront surgery in RPC and BRPC, found a difference in median survival of only 1.4 months (15.7 vs. 14.3 months, *p* = 0.025) (25). However, the 5-year overall survival rate was 20.5% with neoadjuvant chemoradiotherapy and 6.5% with upfront surgery. The survival benefit of neoadjuvant chemoradiotherapy was consistent across the pre-specified subgroups, including RPC and BRPC. Although only currently available as abstract, Unno et al. found a median overall survival of 36.7 months for neoadjuvant gemcitabine and S1, and 26.6 months for upfront surgery in 362 patients with RPC and BRPC (*p* = 0.015) (20). Both groups received adjuvant S1. All trials in the meta-analysis by van Dam et al. included a neoadjuvant gemcitabine-based chemotherapy arm, and none of these two trials used adjuvant FOLFIRINOX (10). Of note, most ongoing RCTs compare neoadjuvant FOLFIRINOX with upfront surgery and schedule patients in both arms to adjuvant FOLFIRINOX (Table 1).

## Optimal Duration of Neoadjuvant Chemotherapy

Ongoing or completed RCTs in RPC and BRPC include different perioperative chemotherapy regimens, and the duration of neoadjuvant and adjuvant chemotherapy differs between studies (Table 1). Most trials perform surgical resection between short-course neoadjuvant therapy (SNT) and adjuvant therapy. The optimal duration of perioperative systemic chemotherapy is defined as 6 months based on the results from RCTs of adjuvant chemotherapy in patients undergoing upfront surgery (4, 6–9). Accordingly, 10 of 11 RCTs administer neoadjuvant chemotherapy for 2–4 months, followed by adjuvant chemotherapy for 2–4 months following a successful surgical resection. Only PREOPANC-2 does not include adjuvant chemotherapy as part of the treatment sequence in the experimental arm, but complete multimodal treatment after eight cycles (4 months) of FOLFIRINOX and surgical resection (18).

The added value of radiotherapy following neoadjuvant chemotherapy in patients with RPC and BRPC is unclear. Radiotherapy, in the treatment of RPC and BRPC, is more common in the United States (2). Only PREOPANC-1/2 and ESPAC-5F have included radiotherapy in the neoadjuvant arm (17–19). In recent meta-analysis, a radiotherapy following neoadjuvant FOLFIRINOX was associated with an improved R0 resection rate as compared with neoadjuvant FOLFIRINOX alone, but a difference in survival could not be demonstrated (26).

For BRPC, neoadjuvant therapy has been established as routine practice in many centers and countries (2, 27). In a recent systematic review and patient-level meta-analysis of 24 studies comprising 313 patients with BRPC treated with neoadjuvant FOLFIRINOX, the median number of neoadjuvant FOLFIRINOX cycles ranged from 4 to 9 (2–5 months) (28). In a large international, multicenter cohort study of 536 patients, including 243 (48.4%) with RPC and 208 (41.4%) with BRPC, underwent pancreatectomy after the neoadjuvant FOLFIRINOX. The median number of neoadjuvant FOLFIRINOX cycles was 6 (3 months) (29). To date, no data exist to define whether a higher number of neoadjuvant chemotherapy cycles is associated with improved survival.

Some centers suggest a role for total neoadjuvant therapy (TNT), also termed the surgery-last approach for RPC and BRPC (24, 30–32). Kim et al. recently published the Wisconsin experience with TNT in patients with operable pancreatic cancer and included all patients who initiated neoadjuvant therapy with surgical/curative intent (24). The study reviewed 541 patients including 226 (42%) with RPC and 315 (58%) with BRPC. The TNT was administered to 89 (16%) of the patients and 452 (84%) received SNT. Both groups underwent neoadjuvant chemotherapy for a minimum of 2 months in SNT and 4 months in TNT, followed by chemoradiotherapy for 5.5 weeks (Table 1). Patients who were treated with TNT received a median duration of 5.5 months of non-surgical therapy compared with 4 months for patients treated with SNT (*p* < 0.01). The study suggests that patients who can tolerate SNT would likely benefit from TNT. The rate of completion of all intended neoadjuvant therapy and surgery was not statistically different between the two groups (SNT: 71% and TNT: 72%, *p* = 0.90). Thus, TNT did not seem to risk the loss of a window of operability, which is an important finding when TNT is extended to patients with RPC. Thus, most RCTs in RPC administer SNT for 2–3 months; PREOPANC-2/3 and ALLIANCE-021806 have prolonged the duration of neoadjuvant therapy and administered neoadjuvant mFOLFIRINOX for 4 months in the experimental arm (12, 18, 19). In PREOPANC-3 and ALLIANCE-021806, additional adjuvant chemotherapy is administered for 2 months in the experimental arm. When giving TNT, restaging with CT is performed after the first 4 cycles (2 months) of mFOLFIRINOX, and patients with treatment response or stable disease according to Response Evaluation Criteria in Solid Tumors (RECIST) criteria are scheduled for an additional four cycles of neoadjuvant mFOLFIRINOX (18). According to the PREOPANC-2 protocol, discontinuation of neoadjuvant chemotherapy after 2 months was recommended if CT showed metastatic disease or local tumor progression determined by RECIST criteria (i.e., at least a 20% increase in the longest diameter of the tumor). Accordingly, patients with local tumor progression proceeded to surgical exploration after discontinuation of chemotherapy, unless a CT scan showed metastatic or locally advanced, unresectable disease (18). The risk of development of distant metastases during neoadjuvant therapy has been reported to be up to 15% (23, 33). This rate equals the occurrence of early disease recurrence in patients with RPC undergoing upfront surgery (34, 35). Patients with pancreatic cancer, who develop early distant metastases after upfront major pancreatectomy, has probably undergone the stress of pancreatectomy for no oncologic gain. However, the risk of local tumor progression during neoadjuvant chemotherapy is a major concern, especially if the patients are losing a curative surgical window. The PREOPANC-2/3 and ALLIANCE-021806 will give important information about the risk of local tumor progression during TNT. It is reasonable that patients with RPC experiencing local tumor progression after 2 months of neoadjuvant chemotherapy are offered discontinuation of chemotherapy and surgical resection in these trials. An alternative could be to switch chemotherapy regimens or give additional radiotherapy. In the TNT study by Kim et al., the chemotherapy regimen was changed if serum CA19-9 levels did not decrease or if the tumor appeared to increase on imaging after the first 2 months of neoadjuvant chemotherapy. However, details on the number of patients who had to change the chemotherapy regimen, or if any patients with local tumor progression discontinued chemotherapy and proceeded to surgical exploration, are not given in that paper (24).

Patients undergoing neoadjuvant chemotherapy may benefit from a chemotherapeutic switch before resection if the response evaluation shows tumor progression (32, 36). The TNT may allow for the switch of chemotherapy that is not possible to implement during SNT. Alva-Ruez et al. recently published the Mayo experience with a chemotherapeutic switch in 468 patients with BRPC and patients with locally advanced pancreatic cancer (LAPC), undergoing first-line chemotherapy without the development of metastatic disease on initial restaging examinations (36). After a median of six cycles of neoadjuvant chemotherapy, 70% (329/468) continued with the first-line chemotherapy regimen after restaging and subsequently underwent surgical resection. The remaining 30% (139/468) of patients underwent chemotherapy switch due to radiological or biochemical progression, no objective response, or toxicity/intolerance. Of patients who underwent chemotherapeutic switch, 72% were able to proceed to curative-intent surgical resection. Although these patients, probably, were highly selected and this was a non-randomized study, the strategy of the chemotherapeutic switch is interesting to implement in future RCTs on SNT vs. TNT in RPC and BRPC.

Kim et al. showed that patients undergoing TNT had improved histologic response [SNT vs. TNT; complete response 4 vs. 8%, near-complete response 15 vs. 16%, partial response 53 vs. 76%, no response 26 vs. 2%; (*p* < 0.01)] (24). A prognostic and reproducible system for histological tumor response that is scoring in pancreatic cancer may improve comparisons between different trials. More extensive tumor response in one treatment group could indicate a superior treatment effect, and histopathological tumor response scoring could function as a potential surrogate outcome in studies comparing the effectiveness of various durations of neoadjuvant chemotherapy (37).

## Evaluation of Treatment Response

During neoadjuvant therapy, the patients are evaluated for treatment toxicities and objective clinical, radiologic, biochemical, or metabolic responses, typically every 2 months (24, 36). If TNT is given, a proper evaluation of treatment response is especially required. However, there is a lack of efficient biomarkers or imaging techniques to monitor treatment responses during neoadjuvant chemotherapy in pancreatic cancer (38, 39). Current assessments using CT or CA19-9 show only modest objective response rates during neoadjuvant chemotherapy (38). In resected patients, low post-treatment CA19-9 levels are independently associated with a histopathologic major response and normalization of CA19-9, rather than the magnitude of change, and has shown to be a strong prognostic marker for a long-term survival (40, 41). A recent systematic review of 17 eligible studies with complete information on CA19-9 response during neoadjuvant therapy found that post-neoadjuvant CA19-9 response >50% or CA19-9 normalization was related to a more promising overall survival, suggesting that optimal CA19-9 response may be a suitable prognostic index to guide treatment decisions (42).

Katz et al. have shown that radiographic downstaging is rare after neoadjuvant therapy, and RECIST response evaluated on CT was not considered as an effective treatment endpoint for patients with BRPC, since only 12% had partial radiographic response (43). Accordingly, patients with RPC and BRPC undergo pancreatectomy after neoadjuvant therapy in the absence of metastases or a locally advanced and unresectable disease. Perri et al. assessed radiographic and serologic measures of treatment responses associated with first-line chemotherapy with FOLFIRINOX or gemcitabine/nab-paclitaxel in 485 consecutive treatment-naive patients with localized pancreatic cancer (44). Among the 280 matched patients, RECIST partial response was more common among patients treated with FOLFIRINOX than with gemcitabine/nab-paclitaxel (19 vs. 6%; *p* = 0.001), whereas no differences were observed in the median change in tumor volume, the rate of local tumor downstaging, or the CA19-9 levels. In recent studies on TNT, a radiographic downstaging of 28% has been found, maybe due to the use of modern chemotherapy regimens (32, 38). Post-chemotherapy metabolic responses based on positron emission tomography (PET/CT or PET/MRI) metabolic imaging as surrogates of pathologic response is currently used in some centers to evaluate treatment response (32, 45, 46). Metabolic response by PET/CT or PET/MRI may be a more sensitive measure of tumor response than traditional CT or MRI and should be evaluated in future clinical trials (47). Criteria to be considered in the evaluation of treatment response during neoadjuvant therapy in pancreatic cancer are presented in Table 2.

**Table 2:** Criteria to be considered in the evaluation of treatment response during neoadjuvant therapy in pancreatic cancer.

**Response evaluation**	**Criterias**	**Comment**
CA19-9	CA19-9 decrease or normalization	CA19-9 response >50% or CA19-9 normalization is related to improved overall survival (40–42)
CT	RECIST response (≥30% decrease in two-dimensional measurement of maximum tumor diameter)	Radiographic response expected in 12–28% of patients, but radiological downstaging is not associated with overall survival (32, 43)
PET/CT	Metabolic tumor response [reduction in standardized uptake value (SUV_max_ and SUV_peak)_ and metabolic tumor volume (MTV)]	Reduction in SUV_max_, SUV_peak_ and MTV indicates improved overall survival (32, 46, 47)
PET/MRI	Metabolic tumor response [reduction in standardized uptake value (SUV_max_ and SUV_gluc)_ and complete metabolic response]	Reduction in SUV_max_ and SUV_gluc_, and complete metabolic response associated with pathological response and improved overall survival (45, 47)

## Impact of Adjuvant Chemotherapy After Neoadjuvant Chemotherapy

The potential benefits of adjuvant chemotherapy after neoadjuvant therapy are not well-defined (29). The choice of adjuvant regimen is often based on response seen to neoadjuvant therapy and other clinical considerations, such as performance status and patient tolerability (2). Whether continuation of the same regimen is worthwhile in case of poor histopathological response remains to be established. Based on data from the National Cancer Data Base, Kamarajah et al. showed that adjuvant chemotherapy, following neoadjuvant chemotherapy and resection, was associated with improved survival, even in R0 and N0 disease (48). However, van Rossel et al. showed that adjuvant chemotherapy after neoadjuvant FOLFIRINOX and resection was associated with improved survival only in patients with N1/N2 disease (29). This is by the treatment strategy given by Kim et al., where patients who received SNT, in general, were recommended to receive additional adjuvant therapy in the absence of a very favorable pathology report (e.g., a near-complete or complete response and N0) or inadequate recovery from surgery (24).

## Refinement of Perioperative Chemotherapy in the Era of Precision Medicine

The personalized medicine approach in pancreatic cancer still needs to remove some barriers to be effective in clinical practice (11, 49). The patients with RPC and BRPC currently receive a one-size-fits-all treatment strategy based on results from RCTs on adjuvant and neoadjuvant therapy (4, 6–9, 16, 19). Ongoing RCTs also follow the principle of one-size-fits-all. However, a broad heterogeneity is observed in survival and response to neoadjuvant therapy with FOLFIRINOX and gemcitabine/nab-paclitaxel in patients with RPC and BRPC. The FOLFIRINOX has been associated with higher rates of RECIST partial response and subsequent pancreatectomy than gemcitabine/nab-paclitaxel, but the overall survival associated with these regimens are similar (16, 44). A neoadjuvant strategy should select and use the most effective and clinically tolerable chemotherapy regimen (36). Currently, a selection of combination regimen is based entirely on clinical criteria, and no biomarkers exist to guide the choice of chemotherapy in RPC and BRPC. A better patient stratification is needed to guide personalized treatment strategies and to prevent chemotherapy-related toxicity to improve outcomes. Interestingly, a pancreatic cancer patient-derived organoid library obtained gene expression signatures of chemosensitivity that predicted improved response to chemotherapy (50). Hopefully, this methodology may identify genomic, transcriptomic, and therapeutic profiling to guide the choice of chemotherapeutics and enable stratification of patients. By this approach, patients may rapidly achieve clinical benefits while more tailored treatments can be developed for each patient.

Liquid biopsies enable the collection of repeated samples during neoadjuvant therapy and can help to stratify patients to the most suitable treatment and to monitor treatment response. Several biomarkers, including circulating tumor cells (CTCs), circulating tumor DNA (ctDNA), cell-free RNA (cfRNA), and exosomes are explored, with the potential to be applied to the clinical setting (51). Several ongoing clinical trials explore the use of liquid biopsies for early diagnosis or predictive and prognostic purposes in pancreatic cancer (51). Gemenetzis et al. showed that patients who had received neoadjuvant treatment had significantly lower numbers of CTCs across all phenotypes compared with a chemo-naive cohort undergoing upfront surgery (52). Moreover, Bernard et al. found that monitoring of exoDNA during neoadjuvant therapy provided predictive information on treatment response in patients with localized pancreatic cancer (53). In a recent multicenter study of 504 patients receiving neoadjuvant FOLFIRINOX or gemcitabine/nab-paclitaxel ± radiation, followed by pancreatectomy, 104 patients received TNT that was associated with an increased rate of major (complete/near-complete) pathologic response and overall survival (31). However, pathologic complete response (pCR) does not tell the whole story. Interestingly, Yin et al. found that somatic mutations, CTCs, and ctDNA existed even in patients with pancreatic cancer with pCR to neoadjuvant therapy, and proposed a new concept of regression assessment by combining genomic analysis of resected specimens and liquid biopsy data, namely, molecular complete response (mCR) (54). Efforts to develop predictive and therapy-response circulating biomarkers are underway, and will represent important tools for monitoring patients, supporting clinicians, and guiding treatment decisions in the future.

More patients with pancreatic cancer might benefit to undergo tumor molecular profiling or receive targeted therapies (55). Some novel attempts to incorporate tumor profiling into the treatment of pancreatic cancer have been published. A retrospective analysis of the Know Your Tumor registry trial showed that patients who had actionable molecular alterations had benefited from receiving a matched therapy (56). However, only a small percentage of all pancreatic cancer patients have mutations that can be targeted. Best-in-class examples of potentially targetable genetic alterations were found in about 8% of the patients; Poly (ADP-ribose) polymerase (PARP) inhibitors targeting germline Breast cancer gene (BRCA)1/2 mutations, Tropomyosin receptor kinase (TRK) inhibitors for Neurotrophic tyrosine receptor kinase (NTRK)1/2/3 fusions, and immune checkpoint inhibitors for Mismatch repair (MMR)-deficient or Microsatellite instability high (MSI-H) tumors (56). Although most treatment recommendations are largely based on data from treatment of metastatic and locally advanced pancreatic cancer, some of these findings could be explored in RPC and BPRC in future clinical trials. The European Society of Medical Oncology (ESMO) recommends clinical research centers propose multi-gene sequencing to patients with advanced pancreatic cancer in the context of molecular screening programs, for patients to get access to innovative drugs (57). If multigene sequencing is not carried out, the ESMO currently recommends that detection of MSI-H and NTRK fusions should be done using cheaper standard methods. In BRPC, the NCCN currently recommends core tumor biopsy to be performed at the time of diagnosis to obtain adequate tissue for possible ancillary studies (2). However, at present, there is limited evidence to recommend specific neoadjuvant regimens other than FOLFIRINOX and gemcitabine/nab-paclitaxel off-study. The NCCN suggests that testing for actionable somatic findings with fusions [Anaplastic lymphoma kinase (ALK), Neuregulin-1 gene (NRG1), NTRK, ROS proto-oncogene 1 (ROS1)], mutations [Proto-oncogene B-Raf (BRAF), BRCA1/2, Human epidermal growth factor receptor 2 (HER2), Kirsten rat sarcoma viral oncogene homologue (KRAS), Partner and Localizer of BRCA2 (PALB2)], and MMR deficiency must be considered in metastatic or locally advanced, unresectable disease (2). For known BRCA1/2 or PALB2 mutations, NCCN recommends gemcitabine/cisplatin or (m)FOLFIRINOX. For MSI or MMR tumors, pembrolizumab is considered as an option, whereas for NTRK gene fusion-positive disease, larotrectinib or entrectinib may be considered (2). At present, targeted therapy is probably most important to explore in patients with LAPC to achieve downstaging and surgical resection or in metastatic disease. However, given the risk of progression during neoadjuvant therapy and high risk of recurrence after surgical resection in patients with RPC and BRPC, tumor/somatic gene profiling to identify uncommon mutations should also be considered in these patients at the time of diagnosis, so that tailored treatments can be considered early in patients with progressive disease.

## Conclusion

Most RCTs in RPC and BRPC have explored SNT, but TNT is now evaluated in several ongoing trials. The chemotherapeutic switch could be incorporated into neoadjuvant treatment sequencing in future trials. Components of an objective response include clinical, biochemical (CA19-9 decrease), radiologic (decreased tumor size/less vascular involvement), or metabolic responses (decreased tumoral PET avidity/viability). In the future, biomarker hypothesis-driven clinical trials and better-circulating biomarkers (CTCs, ctDNA, cfRNA, and exosomes) and imaging techniques (PET/CT, PET/MRI) to evaluate treatment responses are needed to guide personalized therapy and improve outcomes for patients with pancreatic cancer.

## Author Contributions

KJL contributed to conception and design of the review, wrote the first draft of the manuscript and revised, read, and approved the submitted version.

## Funding

This work was supported by the South-Eastern Norway Regional Health Authority (grant number 2019029).

## Conflict of Interest

The author declares that the research was conducted in the absence of any commercial or financial relationships that could be construed as a potential conflict of interest.

## Publisher's Note

All claims expressed in this article are solely those of the authors and do not necessarily represent those of their affiliated organizations, or those of the publisher, the editors and the reviewers. Any product that may be evaluated in this article, or claim that may be made by its manufacturer, is not guaranteed or endorsed by the publisher.
